# Genomic Predictors of Asthma Phenotypes and Treatment Response

**DOI:** 10.3389/fped.2019.00006

**Published:** 2019-02-05

**Authors:** Natalia Hernandez-Pacheco, Maria Pino-Yanes, Carlos Flores

**Affiliations:** ^1^Research Unit, Hospital Universitario N.S. de Candelaria, Universidad de La Laguna, Santa Cruz de Tenerife, Spain; ^2^Genomics and Health Group, Department of Biochemistry, Microbiology, Cell Biology and Genetics, Universidad de La Laguna, Santa Cruz de Tenerife, Spain; ^3^CIBER de Enfermedades Respiratorias, Instituto de Salud Carlos III, Madrid, Spain; ^4^Genomics Division, Instituto Tecnológico y de Energías Renovables, Santa Cruz de Tenerife, Spain

**Keywords:** admixture mapping, asthma, genomics, genome-wide association study, multiomics, personalized medicine

## Abstract

Asthma is a complex respiratory disease considered as the most common chronic condition in children. A large genetic contribution to asthma susceptibility is predicted by the clustering of asthma and allergy symptoms among relatives and the large disease heritability estimated from twin studies, ranging from 55 to 90%. Genetic basis of asthma has been extensively investigated in the past 40 years using linkage analysis and candidate-gene association studies. However, the development of dense arrays for polymorphism genotyping has enabled the transition toward genome-wide association studies (GWAS), which have led the discovery of several unanticipated asthma genes in the last 11 years. Despite this, currently known risk variants identified using many thousand samples from distinct ethnicities only explain a small proportion of asthma heritability. This review examines the main findings of the last 2 years in genomic studies of asthma using GWAS and admixture mapping studies, as well as the direction of studies fostering integrative perspectives involving omics data. Additionally, we discuss the need for assessing the whole spectrum of genetic variation in association studies of asthma susceptibility, severity, and treatment response in order to further improve our knowledge of asthma genes and predictive biomarkers. Leveraging the individual's genetic information will allow a better understanding of asthma pathogenesis and will facilitate the transition toward a more precise diagnosis and treatment.

## Introduction

Asthma is a complex respiratory disease characterized by inflammation and reversible obstruction of the airways (1) that can lead to diverse symptoms such as wheeze, breathlessness, chest tightness, and cough (2). Asthma affects approximately 350 million people from all age groups worldwide (3) and causes around 350,000 deaths per year (4). Although asthma is a lifelong disease, it is considered the most common chronic condition in children and young adults (5, 6), where symptoms are usually more severe (7, 8).

A significant global burden has been attributed to asthma, which is mostly driven by direct economic costs on health care systems (9) and indirect social and economic consequences due to substantial productivity loss (10). In this regard, asthma represents one of the most important pulmonary diseases (11). However, wide differences in asthma prevalence have been estimated among countries and populations, ranging from 1.5 to 15.6% (12, 13), and also among ethnic groups within countries (14). These differences could be a result of complex interactions among environmental and genetic factors (15, 16).

Several studies support a large genetic contribution to asthma predisposition, known as heritability (17, 18), with estimates of as much as 55–74% in adults (19, 20), and almost reaching 90% in children (21). In order to elucidate the genes underlying asthma pathogenesis, several genetic approximations have been performed (22). The initial studies were linkage analyses, which are based on small panels of informative markers across the genome that were determined in multigenerational families with multiple affected individuals to allow the identification of the markers that were more frequently co-inherited with the disease (23). After a genomic region is linked to a disease, this could be followed-up using positional cloning, or the genes contained therein might serve as candidate regions for association studies in outbred population samples (23). Although the use of this approach for over 20 years allowed the identification of as few as eight asthma genes [reviewed in (24–26)], it was recognized a lack of power of this approach for detecting small effect sizes of the risk variants (27).

The use of linkage analysis decreased as it was a progressive development and use of candidate-gene association studies (23, 27). The latter were extensively used during the last two decades, mostly to refute or confirm the implication of a single biological candidate gene at a time (28), mainly by comparing the allele frequencies of a small set of single nucleotide polymorphisms (SNPs) near or within the gene of interest among asthma cases and control subjects without the disease (29).

Although candidate-gene association studies largely increased the resolution of genetic studies of asthma compared to linkage analyses, they also complicated the interpretation of overall results. The main reasons for that were that most studies have included small sample sizes and have tested a reduced number of genetic variants, which greatly decreases the power to detect significant associations. Most importantly, replication of findings in, at least, an independent study was not a standard practice. As a consequence, failure in the attempt to consistently replicate the findings in independent populations was common (23, 29). Given these criticisms, its use has progressively decreased while advances in high-throughput polymorphism genotyping platforms were occurring, leading to continuous reductions in costs and the development of key analysis methods to allow much denser genomic scans (29). These advances opened the way for genome-wide association studies (GWAS), which now allow a simultaneous exploration of hundreds of thousands of SNPs across the genome, most commonly determined in samples from unrelated cases and controls (23, 29). The main advantage of this hypothesis-free approach is the ability to detect mild effects of disease genes without any previous knowledge of the condition (23, 29). On the other side, performing GWAS could be challenging as they usually require large sample sizes and the coverage of the largest number of variants as possible to reach enough statistical power to detect significant associations with asthma (29).

Vicente et al. recently discussed the GWAS that were published from the first one in 2007 (30) until the end of 2016 (31), revealing a total of 39 common SNPs independently associated with asthma risk (22). In this review, we aimed to update the main findings of the genomic studies of asthma, treatment response and the overlap of this disease with other allergic conditions performed between 2016 and 2018. Additionally, we discuss the direction of the new generation of genetic studies of asthma to cover the unexplored variation and the forthcoming integrative omics approaches to continue disentangling the genetic predictors of asthma.

## Genome-Wide Association Studies

A search on the NHGRI-EBI GWAS Catalog (32) and on PubMed records revealed that 15 GWAS of asthma and related traits had been published after the period reviewed by Vicente et al. (22), between 1st May 2016 and 19th September 2018 (Supplementary Table 1).

Asthma was defined by a physician diagnosis in most of the studies. However, some GWAS also considered other asthma definitions, such as the presence of symptoms or the prescription of any asthma medication, among others. Four GWAS focused on children (33–36) and five on adults (37–41), whereas another five attempted to identify common genetic factors between childhood and adulthood asthma (42–46). Across the 15 GWAS of asthma and related traits reviewed, the largest sample sizes were attained by those focusing on the genetic overlap of asthma and allergic diseases. The largest one included 360,838 individuals (180,129 cases and 180,709 controls) and aimed to disentangle the common genetic basis of asthma, hay fever and eczema in asthmatic children from European populations (45). The smallest comprised 949 individuals and it was focused on a highly specific phenotype, the response to asthma treatment with short-acting β_2_ agonists (SABA) (36) (Supplementary Table 1).

Although there is an increasing trend to include multiethnic populations in genomic studies of asthma, an underrepresentation of non-European populations is still pervasive (47, 48). In fact, the vast majority of GWAS performed between 2016 and 2018 focused on patients of European ancestry (33, 34, 39, 41–43, 45, 49), presenting a particularly poor representation of Asians and Africans-admixed populations.

In total, 451 genetic variants, including short insertions/deletions and SNPs, were reported as risk factors for asthma and related traits by the GWAS of the last 2 years. From these, 319 SNPs clustered at 167 loci that reached genome-wide significance at a threshold of *p* < 5 × 10^−8^ or *p* < 3 × 10^−8^ in the discovery or replication phases and/or after performing a meta-analysis with the results from both stages. Among these, 68 were revealed as novel asthma loci, whereas 99 had been previously associated with asthma or any allergic diseases.

In the sections below, we summarize the main findings of these GWAS, distinguishing among those that focused on asthma susceptibility; treatment response; gene-environment interactions and the overlap among asthma and allergic disorders.

### Asthma Susceptibility

Eight GWAS evaluated the association with asthma (33, 35, 37, 38, 42–44, 46) (Supplementary Table 1), although only four studies revealed genome-wide significant associations (38, 43, 44, 46). These validated the association of 14 loci previously associated with asthma susceptibility (Table 1).

**Table 1:** Summary of the most significant variants identified by the genome-wide association studies of asthma susceptibility.

**SNP^**a**^**	**Chr. region^**b**^**	**Position^**c**^**	**Nearest gene (s)**	**Effect allele**	**OR^**d**^, ^**e**^**	***p*-value^**e**^**	**References**
rs1420101	2q12	102957716	*IL1RL1*	T	1.12	3.90 × 10^−21^	(46)
rs10455025	5q22	110404999	*TSLP*	C	1.15	9.40 × 10^−26^	
rs20541	5q31	131995964	*IL13*	G	0.89	5.00 × 10^−16^	
rs7705042	5q31	141492419	*NDFIP1*	A	1.09	7.90 × 10^−9^	
rs9272346	6p21	32604372	*HLA-DQA1*	A	1.16	5.70 × 10^−24^	
rs2325291	6q15	90986686	*BACH2*	A	0.91	2.20 × 10^−12^	
rs992969	9p24	6209697	*IL33*	G	0.86	7.20 × 10^−20^	
rs7927894	11q13	76301316	*LRRC32*	T	1.10	2.20 × 10^−14^	
rs167769	12q13	57503775	*STAT6*^f^	T	1.08	3.90 × 10^−9^	
rs2033784	15q22	67449660	*SMAD3*	G	1.10	7.40 × 10^−15^	
rs2952156	17q12	37876835	*ERBB2*	G	0.87	2.20 × 10^−30^	
rs17637472	17q21	47461433	*ZNF652-PHB*	A	1.08	6.60 × 10^−9^	
rs200567451	17q21	37902883	*GRB7*^f^	G	NA	5.10 × 10^−9^	(44)
rs12946510	17q21	37912377	*GRB7-IKZF3*	T	NA	5.54 × 10^−13^	
rs907092	17q21	37922259	*IKZF3*	A	NA	8.70 × 10^−14^	
rs36095411	17q21	38031865	*ZPBP2*	G	NA	5.32 × 10^−14^	
rs35569035	17q21	38035624	*ZPBP2-GSDMB*	T	NA	6.36 × 10^−14^	
rs9303279	17q21	38073968	*GSDMB*	C	NA	8.21 × 10^−14^	
rs8076131	17q21	38080912	*ORMDL3*	A	NA	5.19 × 10^−13^	
rs7221814	17q21	38089717	*ORMDL3-LRRC3C*	G	NA	9.37 × 10^−11^	
rs3095318	6p21	31088145	*PSORS1C1*	NA	1.42	1.61 × 10^−11^	(38)
rs1776883	6p21	34156444	*GRM4*^f^	NA	0.80	5.29 × 10^−9^	
rs72721166	9p22	27304548	*EQTN*^f^	NA	1.82	3.83 × 10^−9^	
rs75446656	10q21	65100016	*JMJD1C*^f^	NA	2.64	3.60 × 10^−8^	
rs36080042	10q21	65426785	*REEP3*^f^	NA	2.62	4.70 × 10^−8^	
rs62067034	17q21	38063738	*GSDMB*	T	NA	3.55 × 10^−12^	
rs9303277	17q21	37976469	*IKZF3*	T	1.31	1.43 × 10^−14^	(43)
rs11557467	17q21	38028634	*ZPBP2*	T	1.32	3.29 × 10^−15^	
rs2290400	17q21	38066240	*GSDMB*	C	1.31	2.55 × 10^−20^	
rs4795405	17q21	38088417	*ORMDL3*	T	1.26	1.90 × 10^−15^	

a Only the most significant variants per loci and study are included.

b Chromosomal region.

c Positions based on GRCh37/hg19 build.

d Odds ratio for the effect alleles.

e Association results of the meta-analysis.

f *Novel locus (no previous evidence of association with asthma). NA: not available*.

The well-known 17q21 asthma locus (50) has been the most replicated signal, although the main driver of this association has not been disentangled to date (43). The gene encoding the zona pellucida-binding protein 2 (*ZPBP2*) has been revealed as a common locus of both childhood and adulthood asthma by several studies, supported by the association of several intronic SNPs as well as variants located within the intergenic region of *ZPBP2* and *GSDMB* (43, 44). A SNP located at the promoter region of *ZPBP2* (rs11557467) showed the most significant association after performing a meta-analysis in 13,556 children and adults from several European populations (43). The risk allele was associated with asthma susceptibility (OR for the T allele = 1.32, *p* = 3.29 × 10^−15^) (43) and was also replicated in Latinos/Hispanics (44). This variant was previously evidenced to be a putative site with allele-specific nucleosome occupancy in patients with asthma (51). Similar results were found for *GSDMB*, with a shared signal between both European (min *p* = 2.55 × 10^−20^) (43) and Latino/Hispanic populations (min *p* = 8.21 × 10^−14^) (44). Furthermore, the association of *ORMDL3* with asthma was validated in Latinos/Hispanics (min *p* = 1.90 × 10^−15^) (44), which have been also extensively associated with asthma across different populations (30, 52, 53) (Table 1). Interestingly, differences in the expression level of *ZPBP2* and *GSDMB* have been found between European and African populations (54). In fact, early studies had revealed that SNPs associated with asthma co-regulate the expression of *ORMDL3, GSDMB*, and *ZPBP2* in Latinos (54).

A large multiethnic GWAS performed in 23,948 asthma cases and 118,538 controls validated the association of several genes already known to be involved in asthma with functions related to immune response and other activities, such as organogenesis, cellular differentiation and transcriptional modulation, among others (46). The most significant association signal was driven by the SNP rs2952156 located at the Erb-B2 Receptor Tyrosine Kinase 2 (*ERBB2*) gene, whose G allele was associated with protection for asthma (OR = 0.87, *p* = 2.20 × 10^−30^) in ethnically diverse populations (46) (Table 1).

Additionally, 6 loci not previously linked to asthma were identified in European (38), Latino/Hispanic (44) and multiethnic populations (38). In these studies, the *GRM4* gene was the most frequent signal, where a higher number of variants with evidence of association with asthma susceptibility were located (min *p* = 5.29 × 10^−9^) (38). *GRM4* encodes the glutamate metabotropic receptor 4, involved in synaptic neurotransmission and maintenance on normal functions of the central nervous system throughout the regulation of the adenylate cyclase cascade (55), although it has been recently linked to tumorigenesis (56). The *GRM4* gene has been associated with several neurological disorders (57–59) and different types of cancer (56, 60) but, it has not been associated with any asthma-related traits and it has not been implicated in any immune-related function. However, early studies had suggested the potential implication of glutamate receptors on asthma worsening by means of triggering airways inflammation (61).

### Asthma Treatment Response

The most commonly prescribed medication to treat asthma are SABA and inhaled corticosteroids (ICS) (2). Although most asthma patients treated with these medications experience a decrease in their symptoms (62), wide differences in asthma treatment response have been described among individuals and populations (63, 64). These observations suggest that genetics may play a key role in the response to asthma treatment (64, 65). Therefore, the characterization of multiple genetic markers determining therapeutic responsiveness to asthma medications could contribute in the future to identify specific pharmacogenetic profiles. This would enable clinical identification of those asthma patients that respond unsatisfactorily to these treatments or that experience adverse effects (66). Consequently, the burden of asthma could be reduced by implementing personalized asthma management and therapeutic strategies (67).

SABA are the most commonly prescribed relief asthma medication that quickly reduces bronchoconstriction throughout smooth muscle relaxation of the airways (2). Clinical response to this treatment is frequently assessed as bronchodilator response (BDR), which quantitatively measures the change in airway constriction by means of the change in forced expiratory volume in 1 s after SABA administration (68). However, high variability in BDR among individuals and populations has been described, which has been evidenced to be influenced by environmental and genetic factors (69, 70). In fact, it has been estimated that 47–92% of the total variation in BDR could be attributed to the genetic component (71, 72). Recently, a GWAS of BDR was performed in 949 children with asthma from two African American populations (36) (Supplementary Table 1). This revealed the intergenic region of *SPATA31D1* and *RASEF* as population-specific novel loci of BDR in African American children with asthma (rs73650726, β for the A allele = 0.02, *p* = 7.69 × 10^−9^). Moreover, they found the *PRKG1* to be implicated in BDR shared between African Americans and Latinos/Hispanics (min *p* = 3.94 × 10^−8^) (Table 2). This gene encodes a cyclic guanosine monophosphate-dependent protein kinase involved in several biological processes, such as the nitric-oxide signaling pathway (73, 74), which modulates vasodilation in response to β_2_ agonists (75). This fact together with evidence of expression of *PRKG1* in pulmonary tissues suggest this could be a plausible gene of BDR in African-admixed asthmatic children (76).

**Table 2:** Summary results of the genome-wide association studies of asthma treatment response.

**Treatment**	**SNP**	**Chr. region^**a**^**	**Position^**b**^**	**Nearest gene(s)**	**Effect allele**	**Beta^**c**^, ^**d**^**	***p*-value^**d**^**	**References**
SABA	rs73650726	9q21	85152666	*SPATA31D1-RASEF*^e^	A	−3.80	7.69 × 10^−9^	(36)
	rs7903366	10q21	53689774	*PRKG1*	T	1.23	3.94 × 10^−8^	
	rs7081864	10q21	53690331	*PRKG1*	A	1.23	4.94 × 10^−8^	
	rs7070958	10q21	53691116	*PRKG1*	A	−1.24	4.09 × 10^−8^	
Mepolizumab	rs114633080	6q24	145404255	*IFNA14-IFNA22P*^e^	NA	NA	NA	(39)
	rs137893217	6q24	145427162	*IFNA14-IFNA22P*^e^	NA	NA	NA	
	rs78517277	6q24	145465077	*IFNA14-IFNA22P*^e^	NA	NA	NA	
	rs117220641	6q24	145496192	*IFNA14-IFNA22P*^e^	NA	NA	NA	
	rs10811516	9p21	21255049	*IFNA14-IFNA22P*^e^	NA	NA	NA	
	rs10811517	9p21	21256306	*IFNA14-IFNA22P*^e^	NA	NA	NA	

a Chromosomal region.

b Positions based on GRCh37/hg19 build.

c Beta values for the effect alleles.

d Association results of the meta-analysis.

e *Novel locus (no previous evidence of association with asthma). NA: not available*.

Despite the large improvements in asthma therapeutic strategies in the last decades, ICS are still the most effective and commonly prescribed medication to control symptoms and prevent severe exacerbations in asthma patients (2), which consist of the most important outcome in childhood asthma (77). However, a small proportion of the genetic basis of the ICS response has been disentangled (78–80). In the period reviewed, Mosteller et al. performed the unique additional GWAS that has explored the association of genetic variants with ICS response (Supplementary Table 1). This constitutes the first GWAS of ICS response to include non-European patients. Unfortunately, they did not find any significant finding (40).

In addition to the most common types of medications used to treat asthma, there is an increasing number of emerging therapies, including biological treatments. These have been designed to act directly toward specific components of the T-lymphocyte inflammatory response involved in asthma such as, interleukin 5 (IL-5). This mediator is centrally involved in increasing immunoglobulin E levels and blood and bronchoalveolar eosinophilia in severe asthma. Therefore, the inhibition of IL-5 by using monoclonal antibodies could reduce the high levels of eosinophils (81). A few pharmacogenetic studies have recently evaluated the response to asthma therapies with anti-IL-5 monoclonal antibodies, such as mepolizumab (39) (Supplementary Table 1), which has been evidenced to reduce asthma exacerbations rates and enables asthma control (82, 83). Condreay et al. investigated the association of genetic variants with the response to asthma treatment with mepolizumab measured as number of asthma exacerbations, eosinophil count and immunoglobulin E levels in 1,192 asthma patients. Although no variants reached genome-wide significance level (*p* ≤ 5 × 10^−8^), six SNPs at 6p24 and 9p21 showed suggestive associations with mepolizumab response (Table 2) (39).

Unfortunately, despite the large efforts during the last decades, pharmacogenetic findings are still not able to predict clinical outcomes that are directly applied to asthma patients (84). As happened in the past for the asthma field, and although asthma pharmacogenetic studies have started to evolve toward GWAS approaches (64, 85), the main reason could be that most published pharmacogenomic studies continue to be performed using the candidate-gene approach (64, 80, 85).

### Gene-Environment Interactions

Despite the significant contribution of genetic factors on asthma and related traits, a key role of the gene interactions with exposures to a wide variety of environmental factors has been described (15, 16, 86). Among these, early-life exposures demonstrate a high relevance in the prediction of childhood asthma development, including respiratory infections (87), gut and airway microbiome (87, 88), and tobacco smoke exposure (89). Several strategies have been used to identify gene-environment interactions during the last decades (90, 91), but their application has recently emerged in the form of genome-wide interaction studies (GWIS) (91), which are considered a powerful approach to identify novel disease loci that interact with environmental factors (41).

Two GWIS have attempted to identify gene-environment interactions involved in asthma susceptibility since 2016 (34, 41) (Supplementary Table 1). One of them explored for the first time the interaction of genetic variants with traffic-related air pollution, although previous studies used candidate gene approaches (34). Traffic air pollution measured as nitrogen dioxide levels cause deterioration of asthma symptoms by triggering exacerbations and decreasing the lung function (92, 93). This GWIS in European children revealed five loci that were suggestively associated and three of the SNPs were located at *ADCY2*, a known asthma locus (94). The risk alleles at *ADCY2* were also associated with decreased expression of the gene in peripheral blood. Moreover, differential *ADCY2* expression depending on nitrogen dioxide levels was found, suggesting that this gene could have functional implications on asthma under exposure to traffic-related air pollution (34). Similar results were found for a SNP located within the intergenic region of *B4GALT5* and *SLC9A8* (Table 3), which were revealed as novel plausible genes with functional implications on childhood asthma in interaction with nitrogen dioxide exposure (34).

**Table 3:** Summary results of the genome-wide association studies of gene-environment interactions.

						**Discovery phase**		**Replication phase**		
**Environmental exposure**	**SNP^**a**^**	**Chr. region^**b**^**	**Position^**c**^**	**Nearest gene(s)**	**Effect allele**	**OR^**d**^**	***p*-value**	**OR^**d**^**	***p*-value**	**References**
Nitrogen dioxide	rs727432	5p15	7716078	*ADCY2*	G	1.61	6.67 × 10^−5^	1.13	0.016	(34)
	rs4143882	5p15	7717364	*ADCY2*	A	1.61	4.75 × 10^−5^	0.88	0.015	
	rs6886921	5p15	7718539	*ADCY2*	C	1.71	7.03 × 10^−6^	1.12	0.016	
	rs963146	11q14	83423444	*DLG2*^e^	A	0.67	8.61 × 10^−5^	1.12	0.034	
	rs12455842	18q12	32096284	*MOCOS*^e^	C	0.48	6.10 × 10^−5^	1.30	0.010	
	rs1057251	18q12	32102579	*MOCOS*^e^	C	0.50	6.18 × 10^−5^	1.30	9.40 × 10^−3^	
	rs12457919	18q12	32108100	*MOCOS-FHOD3*^e^	A	0.39	5.52 × 10^−5^	1.30	0.012	
	rs12457919	18q12	33854102	*MOCOS-FHOD3*^e^	A	0.39	5.52 × 10^−5^	NA	0.017	
	rs686237	20q13	47804141	*B4GALT5-SLC9A8*^e^	A	1.69	5.43 × 10^−5^	0.89	1.60 × 10^−3^	
Tobacco smoke	rs9969775	9p23	13561933	*MPDZ-NFIB*^e^	A	0.50	7.63 × 10^−5^	0.65	0.020	(41)
	rs5011804	12p12	25441894	*KRAS-LMNTD1*^e^	C	1.50	1.21 × 10^−4^	1.40	0.030	

a Only the variants with evidence of replication are included.

b Chromosomal region.

c Positions based on GRCh37/hg19 build.

d Odds ratio for the effect alleles.

e *Novel locus (no previous evidence of association with asthma). NA: not available*.

Additionally, a GWIS of active tobacco smoking was conducted in 4,057 patients with adulthood-onset asthma of European ancestry (41) (Supplementary Table 1). It is well-known that second-hand smoke exposure to tobacco smoke increases childhood asthma risk during prenatal and postnatal stages (95–99). Although active tobacco smoke has been associated with asthma onset during adulthood (100), it is still unclear how the genetic variation could affect asthma susceptibility in interaction with tobacco smoke exposure in adults (41). The intergenic SNPs rs9969775 (OR for the A allele = 0.50, *p* = 7.63 × 10^−5^) and rs5011804 (OR for the C allele = 1.50, *p* = 1.21 × 10^−4^), which are located at the *MPDZ*-*NFIB* and *KRAS*-*IFLTD1* loci, respectively, showed significant interactions with active tobacco smoking for late-onset asthma. These findings were validated at nominal level in an independent study (41) (Table 3). Although none of these loci showed any functions specifically related to asthma and none were previously associated with asthma or related traits, the SNP rs9969775 was postulated to be involved in the regulation of gene expression in the lung (41).

### Overlap Among Asthma and Allergic Diseases

Given the firm links in the pathogenesis of asthma and other allergic diseases (20, 101), a few studies used this rationale to explore the overlapping genetic architecture among these diseases (45, 49, 102, 103), including two large-scale GWAS published between 2016 and 2018 (45, 49) (Supplementary Table 1).

Ferreira et al. carried out the largest GWAS of asthma and allergic diseases to date (45). They combined data from 360,838 children and adults from 13 different European studies, including 180,129 patients with self-reported or physician-diagnosed asthma, eczema or hay fever, and 180,709 controls (Supplementary Table 1). They reported 136 independent SNPs at 99 loci as genome-wide significant associations (*p* ≤ 3 × 10^−8^) with susceptibility to asthma or any allergic disease (Supplementary Table 2). A total of 86 variants were located at loci that were already associated with at least one of the diseases under study, whereas 50 other SNPs revealed novel loci that were shared by asthma and allergy (Table 4). A high proportion (96%) of these variants showed similar effects between asthma, eczema and hay fever. The most significant variants were located within or near loci with previous evidence of implication on asthma and/or allergic diseases such as, *WNT11-LRRC32, IL18R1, TLR1*, and *HLA-DQA1*, among others. In fact, the SNP rs7936323 of the intergenic region of *WNT11* and *LRRC32* genes showed the strongest evidence of association (OR for the A allele = 1.09, *p* = 2.20 × 10^−63^) (Supplementary Table 2). Altogether, the 136 SNPs identified accounted for 3.2, 3.8, and 1.2% of the total variation of asthma, hay fever and eczema, respectively. These findings partially explain the co-existence of these diseases in many patients (45) (Supplementary Table 2). Interestingly, evidence of potential functional implication on blood and pulmonary tissues was found for many of the SNPs identified. Specifically, they demonstrated that risk variants shared among asthma, hay fever and eczema are involved in the regulation of gene expression in immune response-related signaling pathways, such as in the B and T cell activation (45). These findings confirmed previous evidence (104) suggesting that these biological processes could be among the ones shared between asthma and allergic diseases (45).

**Table 4:** Novel loci of asthma and allergic diseases revealed by meta-analyses published between 2016 and 2018.

**Phenotypes**	**SNP**	**Chr. region^**a**^**	**Position^**b**^**	**Nearest gene (s)**	**Effect allele**	**OR^**c**^, ^**d**^**	***p*-value^**d**^**	**References**
Asthma/hay fever/eczema/rhinitis	rs12743520	1p22	93037112	*PIGX*	C	0.93^e^	3.83 × 10^−8*e*^	(49)
	rs61815704	1q21	152893891	*IVL-SPRR2E*	C	1.14^e^	5.16 × 10^−9*e*^	
	rs1214598	1q24	167426424	*RP11-104L21.2*	G	0.95^e^	5.14 × 10^−11*e*^	
	rs4916533	3q29	196373582	*LRRC33*	C	0.93^e^	1.66 × 10^−8*e*^	
	rs56267605	4q27	123363109	*ADAD1-IL2*	A	1.05^e^	2.56 × 10^−12*e*^	
	rs6461503	7p21	20560996	*ABCB5*	C	1.05^e^	3.19 × 10^−10*e*^	
	rs2169282	9p24	6350235	*TPD52L3*	G	1.09^e^	1.80 × 10^−10*e*^	
	rs12413578	10p14	9049253	*SLC7A10*	C	0.91^e^	1.09 × 10^−14*e*^	
	rs10876864	12q13	56401085	*SUOX*	A	1.05^e^	1.41 × 10^−13*e*^	
	rs9911533	17q21	38775476	*SMARCE1*	T	0.92^e^	9.70 × 10^−16*e*^	
	rs10414065	19q13	33721455	*SLC7A10-CEBPA*	C	0.91^e^	2.63 × 10^−10*e*^	
	rs2766664	20q13	52171241	*RP4-724E16.2*	G	1.08^e^	8.07 × 10^−11*e*^	
	rs10033073	1p36	4775401	*EVI5*	G	1.04	1.20 × 10^−10^	
Asthma/hay fever/eczema	rs1057258	1p36	234115629	*INPP5D*	C	1.05	1.40 × 10^−10^	(45)
	rs10414065	1q21	33721455	*SLC7A10-CEBPA*	C	1.10	6.10 × 10^−18^	
	rs10663129	1q21	141321836	*RASA2*	ACT	1.04	1.10 × 10^−13^	
	rs10760123	1q42	123650534	*PHF19-TRAF1*	T	1.03	5.20 × 10^−9^	
	rs10910095	2p25	2510755	*TNFRSF14-FAM213B*	G	1.04	2.70 × 10^−8^	
	rs11169225	2q12	50345671	*AQP2*	A	1.05	1.20 × 10^−11^	
	rs12440045	3q28	41782684	*RTF1-ITPKA*	C	1.03	4.90 × 10^−10^	
	rs13088318	3q29	101242751	*FAM172BP-TRMT10C*	A	1.03	8.60 × 10^−9^	
	rs13153019	4p16	176782218	*LMAN2-RGS14*	C	1.04	1.30 × 10^−8^	
	rs13403656	4q24	112269127	*BCL2L11-ANAPC1*	A	1.05	2.20 × 10^−8^	
	rs13384448	4q27	228707862	*CCL20-DAW1*	T	1.04	2.80 × 10^−12^	
	rs227275	5p13	103593898	*MANBA*	C	1.03	3.70 × 10^−11^	
	rs17664743	5q22	50253897	*C7orf72-IKZF1*	A	1.04	6.20 × 10^−11^	
	rs250308	5q31	118684297	*TNFAIP8*	T	1.03	4.00 × 10^−9^	
	rs2910162	6p21	159909345	*MIR3142-MIR146A*	G	1.03	2.50 × 10^−9^	
	rs35469349	7p12	128294709	*PTPRK*	A	1.04	2.30 × 10^−10^	
	rs3540	7p21	91045408	*IQGAP1*	G	1.04	3.30 × 10^−11^	
	rs4296977	8q21	77018542	*GSAP*	C	1.06	2.10 × 10^−13^	
	rs4801001	9p24	52336175	*DYNAP-RAB27B*	T	1.03	5.90 × 10^−9^	
	rs4574025	9q33	60009814	*TNFRSF11A*	T	1.03	6.80 × 10^−9^	
	rs4671601	9q34	64836267	*LOC339807*	C	1.04	8.80 × 10^−9^	
	rs4943794	10p14	41173408	*FOXO1*	C	1.04	7.20 × 10^−12^	
	rs5758343	10p14	41816652	*TEF-TOB2*	A	1.05	4.80 × 10^−14^	
	rs55726902	10p14	48196982	*HDAC7*	G	1.05	2.60 × 10^−16^	
	rs4848612	10q24	112388538	*BCL2L11-ANAPC1*	A	1.04	2.30 × 10^−10^	
	rs61192126	11q13	72394852	*LINC00870-RYBP*	T	1.04	8.90 × 10^−11^	
	rs59593577	11q13	95425526	*SESN3-FAM76B*	C	1.05	1.60 × 10^−11^	
	rs6489785	12q13	121363724	*SPPL3-HNF1A-AS1*	T	1.04	1.60 × 10^−15^	
	rs6977955	12q24	28156887	*JAZF1*	T	1.05	7.10 × 10^−13^	
	rs697852	12q24	226914734	*ITPKB*	A	1.04	1.60 × 10^−9^	
	rs7130753	13q22	111470567	*LAYN-SIK2*	C	1.05	7.00 × 10^−15^	
	rs71368508	14q13	4521473	*SMTNL2-ALOX15*	C	1.12	2.00 × 10^−9^	
	rs7137828	14q21	111932800	*ATXN2*	T	1.03	2.20 × 10^−10^	
	rs7207591	14q24	40414862	*STAT5B*	A	1.04	1.40 × 10^−9^	
	rs72033857	14q24	167390671	*RNASET2-MIR3939*	C	1.06	1.20 × 10^−9^	
	rs7214661	14q32	43430696	*MAP3K14-ARHGAP27*	G	1.03	1.20 × 10^−8^	
	rs73205303	15q15	36467830	*RUNX1*	A	1.04	7.90 × 10^−10^	
	rs74847330	15q22	143831599	*KYNU-ARHGAP15*	A	1.05	1.80 × 10^−9^	
	rs75557865	16p13	121652141	*SLC15A2*	G	1.03	1.60 × 10^−8^	
	rs76167968	17q21	35681738	*SFPQ-ZMYM4*	T	1.06	1.30 × 10^−8^	
	rs76081789	17q21	44846426	*SIK1*	T	1.07	1.30 × 10^−8^	
	rs9323612	20q13	75968608	*JDP2-BATF*	A	1.03	8.60 × 10^−9^	
	rs9372120	20q13	106667535	*ATG5*	G	1.04	4.20 × 10^−11^	
	rs9383820	20q13	157419508	*ARID1B*	C	1.04	1.20 × 10^−8^	
	rs9573092	21q22	73627275	*PIBF1-KLF5*	A	1.03	2.70 × 10^−8^	
	rs9989163	22q13	103235012	*RCOR1-TRAF3*	A	1.03	1.90 × 10^−8^	

a Chromosomal region.

b Positions based on GRCh37/hg19 build.

c Odds ratio for the effect alleles.

d Association results of the meta-analysis.

e *Association results of the discovery phase*.

Besides these findings, 29 of the genes identified by Ferreira et al. encode for proteins that are drug targets for several diseases, including allergic and auto-immune diseases. Interestingly, the protective effect of these genes was found to be correlated with the effect of drugs targeting them, attenuating allergy symptoms. These findings suggest that these could be effective targets to treat allergic diseases or asthma and thus, the proteins encoded by these should be prioritized for pre-clinical evaluation (45).

That GWAS was further complemented in a separate study with a gene-based association analysis using an algorithm that was specifically designed to identify shared risk variants among multiple phenotypes. With this approximation, which helps to increase the statistical power to detect novel risk loci, multiple variants near or within each gene are tested rather than focusing on individual SNP tests at a time (103). By relying on the information of SNPs that modify gene expression levels in different tissues and cell types, also known as expression quantitative trait loci (103, 105), they additionally revealed 19 novel risk genes for allergic diseases, which were not revealed by the previous stages of the study (45). Among these genes, nine showed functions that were closely related to well-known mechanisms involved in allergic diseases and asthma. Although further functional validation is needed, these could also represent novel drug targets (103).

Recently, Zhu et al. performed another GWAS in 110,361 Europeans in an attempt to identify genetic variants shared among asthma, hay fever, eczema and rhinitis (Supplementary Table 1). After performing a cross-trait meta-analysis, 38 loci were associated with both asthma and allergic diseases at genome-wide significance level (Supplementary Table 2). These loci were enriched in essential pathways for several tissues, such as skin, lung and whole blood, among others (49). Among these results, seven hits were novel loci that might contribute to the common genetic architecture of asthma and allergic diseases (49) (Table 4). These findings were consistent with the results reported by Ferreira et al. (45). In fact, a high proportion of the loci revealed by Zhu et al. (49) had been previously identified by Ferreira et al. (45). Interestingly, most of them had functions related to immune response, inflammation and epithelium maintenance, such as *HLA-DQB1-AS1, IL1RL1, FLG-AS1*, and *STAT6*, among others (45, 49).

## Admixture Mapping

It has been evidenced that differences in asthma prevalence and severity among populations and ethnic groups could be partly explained by population-specific genetic factors. Alternative genetic scanning methods take advantage of these population specificities that are not frequently explored by means of GWAS approaches (106, 107), which could contribute to further identify asthma genes. One of such approaches is based on the exploration of the variation in genetic ancestry at chromosome-segment level (termed local ancestry) and the correlation with asthma in populations that are the result of a recent historical admixture, an approach most commonly known as admixture mapping (108, 109). Although both GWAS and admixture mapping are based on genome-wide data obtained by means of genotyping platforms, admixture mapping compares local ancestry estimates with the disease or trait (63). With this approach, a much smaller number of comparisons are involved in the scan, which highly increases the statistical power to detect associations compared to a traditional GWAS approach for a given sample size (110). Besides, this approach gives an opportunity for leveraging the specific genetic architecture of admixed populations, which have been largely underrepresented in genetic studies of asthma (111, 112). Admixed populations are characterized by high correlation over large chromosomal regions resulting from the recent admixture process, therefore these populations simplify gene mapping over longer distances (113–116). Moreover, if the trait of interest affects differentially the parental populations of the admixed, large trait differences are expected among admixed individuals, providing increased power to detect novel associations (117). Besides, the particular allelic configurations of the admixed individuals could interact with genetic risks for asthma, mitigating, or enhancing their effects in disease. In fact, causal variants are transmitted in higher proportion from the parental to the admixed populations, which leads to higher prevalence in the latter (112). As a consequence, it is expected that the proportion of the parental ancestry at those loci will vary between asthma cases and control individuals (110, 113), which would be indicative of ancestry-specific genetic risks (63). Combining admixture mapping with the traditional GWAS is a suitable strategy to identify both asthma risks that are ancestry-specific and those that are shared among different ancestry groups (63, 114). Although recently admixed populations are abundant (63), those with African admixture have been the prevalent in the asthma field (69, 109, 115).

There are two major African-admixed populations in the United States: African Americans and Latinos/Hispanics, which show different proportions of ancestry from each parental population (63). However, both show evident genetic footprints of the African admixture (116). Although very simplified, Latinos/Hispanics are usually modeled as descendants of ancient Native American, European and sub-Saharan African populations (63, 117), whereas current African Americans are modeled as descendants of an admixture event between sub-Saharan African and Europeans (63, 110, 118). Interestingly, compared to European Americans, asthma prevalence is higher in these populations, which also show a decreased response to asthma medications (14, 69, 119).

During the last decades, several loci have been associated with asthma and related traits in African Americans and Latinos/Hispanics using admixture mapping analysis as it has been reviewed by Mersha et al. (63) and Hernandez-Pacheco et al. (109). Additionally, two more admixture mapping analysis of asthma susceptibility and treatment response in African-admixed populations had been published by September 2018 (36, 120).

Spear et al. performed a genome-wide exploration in order to identify those genomic regions in which African ancestry is associated with response to asthma treatment with SABA in 949 African Americans. They found that local African ancestry at the 8p11 locus was suggestively associated with BDR in African American children with asthma, though the result did not reach significance level after considering the multiple comparisons (β = 1.49, *p* = 6.34 × 10^−4^) (36).

Additionally, Gignoux et al. revealed that the risk linked to the 18q21 locus in the admixture mapping peak in Latinos/Hispanics was driven by the Native American ancestry (OR = 1.20, *p* = 1.63 × 10^−3^), whereas the European ancestry was protective (OR = 0.86, *p* = 8.35 × 10^−3^), which was validated in an independent Hispanic/Latino population. Interestingly, this peak is located within the intergenic region of *SMAD2* and *ZBTB7C*, none of which have been previously associated with any asthma-related trait, even in GWAS analyzing the same study populations (120–122), suggesting that admixture mapping is a powerful approach to identify novel asthma loci in admixed populations (120). The *SMAD2* gene encodes a cofactor involved in regulation of the growth factor β signaling that has been extensively evidenced to play a key role in asthma (69, 123, 124).

## Asthma Prediction and Translation into the Clinic

In the last 2 years, many asthma genes have been discovered and validated in independent populations, strongly supporting that these are generally involved in asthma pathogenesis. However, the genetic risk factors identified to date only represent a small proportion of total asthma heritability (22, 23, 125). Therefore, despite the uncountable advantages of the GWAS compared to previous strategies (23, 27, 126), there are a number of challenges ahead in order to better understand the genetic architecture of asthma (23, 126, 127).

One of the potential explanations of the current difficulty in explaining a larger proportion of the disease could be the reduced effect size of most genetic risks. Current reference panels and genotyping platforms are mainly designed to capture common genetic variants with are anticipated to show small effects in the disease (23, 114, 125). Therefore, the unassessed genomic variation could help to explain the missing heritability of asthma (114, 128).

Most GWAS of asthma have been limited in terms of low statistical power (23, 125, 127) due to limitations in the study design, mostly due to reduced sample sizes or the underrepresentation of genetically diverse populations, among others (20, 47, 48, 114, 129). A solution to this problem has been attempted in the last years with the emergence of large consortia gathering many asthma studies from different countries around the world (121, 122, 130, 131), which have contributed to increase the representation of patients from multiethnic populations (126, 127). However, this continuous need to increase sample sizes might have also led to heterogeneity in asthma definition by means of combination of samples with different asthma phenotypes. Consequently, this could have contributed to the reduction of the statistical power driven by the dilution of the effect size of association signals among different phenotypes (23, 125, 132–134). Thus, there is an increasing need to accurately characterize asthma patients through classification into homogenous groups (23, 134).

On the other hand, a limited number of large-scale studies have explored the role of gene-environment interactions in asthma despite robust evidence of the important contribution of environmental exposures in asthma susceptibility and severity (15, 16, 86, 135, 136). In fact, it has been evidenced underscoring the significant environmental contribution while designing a GWAS could result in reduced effect sizes (22, 137).

Last but not least, the functional implications of most asthma loci still remain unknown. Therefore, further studies are needed to increase our understanding of the impact of these on genes and cellular function, and their contribution on the molecular mechanisms underlying asthma pathophysiology (22). These have been proposed to be disentangled by means of approaches combining GWAS data with information related to biological pathways or processes (138, 139). Nonetheless, only one GWAS-based pathway enrichment analysis of asthma has been performed to date (140).

Because of all of this, our current knowledge of asthma genetics hampers our capacity to predict disease progression and treatment response, preventing its use in the clinical practice (125, 127). As a result, there is still a long way to use this knowledge and their integration with lifestyle and environment exposures (127, 141, 142) to develop precision medicine strategies for accurate prevention, diagnosis, or treatment of asthma (23).

### Other Omic Studies and Integration of Multiomics

Other omics technologies, apart from genomics, are powerful tools to increase the current knowledge about asthma pathophysiology (143, 144). These are focused on data from a wide variety of biological sources: genomic modifications (epigenomics), gene transcription (transcriptomics), protein levels and chemical modifications (proteomics), endogenous and exogenous metabolites (metabolomics), and the microbiome (metagenomics), among others (23, 126, 127, 145, 146). The application of omics approximations in asthma is still incipient compared to other diseases (23, 147). Still, several studies have been performed in asthma in the last years as it was reviewed elsewhere (23, 127, 148, 149).

To our knowledge, a total of 26 asthma studies using other omics approaches have been published in the last 2 years (Supplementary Table 3). Just like recent GWAS of asthma, these studies have been equally focused on childhood and adulthood asthma since 2016. Moreover, most of them have been carried out in patients of European descent. A total of 18 studies focused on asthma susceptibility or severity (150–167); three focused on the ICS response (168–170); two explored the interactions with environmental factors (171, 172); and three inspected the overlap with other pulmonary diseases (173–175). Nonetheless, other experts have discussed the recent omics advances in asthma in this issue except for those of transcriptomics. Therefore, we focused on summarizing the main findings of studies made using this approach.

Transcriptomics provide a quantitative and qualitative characterization or RNA transcripts (176). These are mainly focused on comparing gene expression levels in cells or tissues under specific controlled conditions in order to identify differentially expressed genes that could have (alone or in combination) functional implications on the disease under study (127, 177). Rapid development of technologies has made possible the near-complete characterization of the transcriptome, first using arrays and later, by means of RNA sequencing, which has greatly promoted the genome-wide exploration of transcriptomic changes in asthma during the last years (23, 127). A number of advantages of transcriptomics studies in asthma have been extensively described (23, 126). In fact, it has been proposed to be an accurate method to characterize pathways contributing to asthma pathophysiology, and the interactions with exogenous and endogenous factors in different sample types such as, blood, sputum or lung tissues, among others (23, 146).

Transcriptomics is a powerful tool to provide or confirm a mechanistic explanation of asthma loci identified by GWAS (23). Eight transcriptomic studies of asthma have been recently performed (Supplementary Table 3). However, most of them were carried out using arrays (157, 158, 167, 170–172) and the majority focused on European populations (157, 163, 170–172) and adults (157, 163, 171, 172, 175). Only three of them explored differential gene expression in children with asthma (158, 167, 170).

The largest transcriptomic study of childhood asthma performed in the last 2 years explored array-based gene expression levels of 133 asthma patients and 11 healthy controls of Asian ancestry (167) (Supplementary Table 3). RNA was extracted from a mixed population of T cells that were isolated from peripheral blood (167). Yeh et al. classified asthma patients into three groups based on 2,048 genes differentially expressed in immune cells. These groups showed distinct inflammatory profiles, including one that clustered the patients with higher neutrophil count and the poorest treatment control, suggesting that these could correspond to those patients with the most severe asthma status. When this group was compared with asthma patients included in other groups, 163 genes were found to be upregulated. Most of these genes encoded proteins involved in glucocorticoid signaling pathway and the immune response, suggesting that this could be an accurate method to classify asthma patients based on transcriptomic data (167). In transcriptomic studies of adulthood asthma, solid or liquid airway samples are frequently used such as, sputum or lung tissues (178, 179). However, clinical procedures to obtain these samples are quite invasive and are especially impractical in children (167). For this reason, peripheral blood has been regarded as the most suitable sample for the studies in children (23, 167).

Asthma diagnosis has classically relied just on conventional clinical guidelines and biomarkers for over decades (180), which are considered very inaccurate due to the wide variety of molecular mechanisms underlying the different asthma phenotypes (181, 182). Is in this respect where integrative approaches that combine complete clinical data and the omics sources could contribute to better characterize the biological processes underlying asthma pathophysiology (182, 183), ultimately helping to define asthma subtypes and to improve the prediction of severity and treatment response.

Multiomics approaches, which incorporate information from different omics levels, have been suggested as a promising strategy to fulfill that purpose (144) as they show an increased predictive capacity (166, 184). Five multiomics studies of asthma and related traits have been performed since 2016. Most of them have combined only a few omics levels (166, 185–188) (Supplementary Table 3).

Forno et al. conducted the largest multiomics study of asthma to date (186). They proposed a novel vertical approach to combine data from different omics levels (genomics, epigenomics, transcriptomics, and proteomics) with clinical information available for 1,127 Latino/Hispanic children, including 618 asthma patients and 509 children without asthma (Supplementary Table 3). Expression of 1,645 genes was associated with cytokine levels in blood, revealing the enrichment of the cytokine signaling pathway. From the 269 genes involved in this pathway, 41 were significantly associated with more than two asthma intermediate phenotypes. As a result, this list was reduced to the *IL5RA* gene, which was found to be the most significant association at the following steps (186). In fact, several transcription factors previously associated with pulmonary diseases showed evidence of association with *IL5RA* (189–191), suggesting that these could be involved in its signaling pathway. Furthermore, low plasma levels of IL-5Rα were found in children with asthma exacerbations, whereas children with earlier age of asthma onset showed increased levels of IL-5Rα, providing firm evidence of implication of *IL5RA* on asthma (186).

Studies as this one suggests that vertical approaches could be a suitable strategy to perform integrative multiomics studies of asthma and even other diseases. However, further validation in independent populations and other complex diseases is needed to confirm the applicability of this method (192). In this respect, a few omics studies of asthma treatment been performed to date, opening an opportunity to identify novel markers that could be applied in the design of precision medicine approaches in asthma and novel therapeutic strategies (67, 193). Although omics approaches have promisingly broken new ground in asthma research, translation into the clinic is still very challenging due to the large amount of information that is obtained.

### Unexplored Genetic Variation in Asthma

Exploring non-coding variation has been also proposed as a promising strategy to disentangle the genetic basis of complex diseases (114) such as, microRNAs (miRNAs). These are short, non-coding and single-strand RNA molecules that interact with different genomic elements and regulate gene expression at transcriptional level (148, 184, 194). Interestingly, these are involved in the regulation of the stability of immune cells and the intensity of inflammation (194). In fact, miRNAs have been proposed as potential non-invasive asthma biomarkers that could be used for asthma diagnosis (195, 196). However, although some authors have suggested the implication of miRNAs on asthma susceptibility, severity, and exacerbations (195, 197, 198), there is a lack of studies that have extensively evaluated their role in asthma (197) and further studies are needed (199).

Structural variation, including copy number variations (CNVs), has been proposed to account for part of the missing heritability of complex traits (114, 200). These involve large chromosomal segments such as, duplications or deletions with consequences on regulation of gene expression (201). It has been reported that CNVs comprise 2% of the total genetic variation (202) with effects on approximately 12% of the human genome (203). This type of variation is enriched within protein-coding genes with functions related to immune response, suggesting its implication on disorders with a significant immunological component such as, asthma (204, 205). Although structural variation has been implicated on asthma, these is an insufficient number of studies to date (206). Some have found strong evidence of association of CNVs with asthma susceptibility (206, 207). Although this type of variation might contribute to an accumulation of mutations and allergic sensitization, leading to an increase of asthma susceptibility, CNVs do not seem to be the initial trigger of asthma development (208).

A substantial proportion of the genetic risk for common diseases could also be explained by variants that are at low frequency in the population (209, 210). The rarer the variant the more likely is for the variant to be population-specific (209, 210). Besides, the pathogenic potential of variants tends to accumulate in the lower range of allele frequency. Therefore, rare variants are more likely to be more structured in populations and to have larger effects on the disease (128, 209). As a corollary, rare variants will be underrepresented in reference datasets and, therefore, remain undetected by traditional GWAS. With this scenario in mind, many rare variants with large effects may be contributing to asthma and allergic diseases (128, 209, 211). However, their study will be only available for now applying sequencing-based methods instead of genotyping arrays. Irrespective of this, endogamous populations are especially appropriate to study the role of rare and low-frequency variation in asthma (210, 212) because rare pathogenic variants are predicted to increase their frequency in these populations (213, 214). Despite this, recent studies have also demonstrated the role of rare variants on recently admixed populations (209), whose inherent characteristics also increase the possibilities to uncover the contribution of rare and low-frequency variants on asthma (215).

Predicted loss-of-function (pLoF) variants, which are likely involved in disrupting protein-coding genes, show significant scientific and clinical interest due to their utility for clinical interpretation of sequencing data. In fact, pLoF variants have been suggested to allow direct identification of causal genes (216) and provide direct mechanistic implications of association effects (217). Although this type of variation has been extensively unexplored for over decades (218), Emdin et al. have recently revealed the potential role of these variants in asthma (217). They found evidence of association of pLoF variants located at well-known *IL33* and *GSDMB* asthma loci with lower risk of both asthma and allergic rhinitis (217). Interestingly, similar results have been found for protein-truncating variants in *IL33* and *GSDMB* (219). These have been predicted to shorten the coding sequence by inducing loss or gain-of-function effects (220). These findings also suggested that exploration of either pLoFs or protein-truncating variants could be another powerful tool for the identification of novel therapies for asthma (217, 219).

As mentioned, the main reason for the scarce evaluation of these types of genetic variation in asthma could be attributed to the fact that research strategies on asthma genetics have focused on using SNP genotyping platforms, which are suboptimal for inferring CNVs, and do not capture rare or pLoF variants (23, 29, 114, 125), as these would be optimally detected by means of sequencing approaches. Given that simultaneous sequencing of millions of small DNA fragments is currently possible at great speed and relatively low cost thanks to large improvements in next-generation sequencing (NGS) technologies (221, 222), the interest on the impact of these types of genetic variation in asthma will continue to rise.

#### Estimation of Polygenic Risk Scores

Another example of the large efforts to try to accelerate the progression toward precision medicine in complex traits is an emerging approach that takes GWAS results as the start point. This consists of stratification of the whole population based on estimates of individual's genetic disease susceptibility measured as polygenic risk scores (PRSs) (223). Just like other complex diseases, the genetic architecture of asthma is polygenic, where many genes contribute to disease development (224). Hence, the overall disease risk could be considered as the result of combined effects driven by common low-risk and rare large-risk variants (225). PRSs are the result of summing up risk alleles from thousand variants revealed by the GWAS (226). Even though most common variants show small effects, combining their effects could explain a significant proportion of the disease variability or at least allow classifying patients into discrete subgroups based on different levels of probabilistic disease risk (223, 226, 227).

Although multilocus profiles of genetic risks for asthma have been constructed using small sets of variants (101), large evaluations of PRSs are lacking for asthma. Nevertheless, previous studies that focused on other complex diseases (228–236) suggest that this approach could be fruitful for asthma. For instance, Khera et al. recently estimated PRSs for five common disorders with major public health impact, including coronary artery disease (236). They found 20-fold greater coronary artery disease risk using a PRSs involving many genetic variants than previous studies based on biomarkers or the mutation panels traditionally used in the clinical practice (236).

Uncountable utilities of PRSs have been demonstrated for the study of common diseases, suggesting its plausibility in a healthcare scenario (227). In fact, it has been proposed that PRS estimation could facilitate the development of accurate preventive, diagnostic and therapeutic strategies (223, 227, 236). Moreover, given the previous evidence of genetic overlap among different diseases (35, 45, 49, 103, 227, 237), an evaluation of individual risks could be assessed simultaneously for multiple traits at a time. This would potentiate the implementation of common therapeutic strategies for different diseases (227, 236). For all these reasons, calculation of PRS has been considered as a feasible approach to translate asthma research findings into healthcare practice for early disease detection (227).

There are many technical, economic, and sociopolitical barriers that should be overcome for the use of PRSs into clinical practice. By one hand, physicians would need additional training to correctly interpret and communicate PRSs to the patients (227). On the other hand, most current PRS estimates are based on loci that were mapped using designs with an overwhelming number of European patients. Therefore, their generalizability in populations of non-European ancestries are questionable (223, 236, 238) due to the large differences in terms of effect sizes, allele frequencies and linkage disequilibrium patterns. Besides this, most PRSs have been estimated in adults. Therefore, an evaluation of their usefulness in other age groups will be needed.

For asthma, the major limitation of PRSs is related to the reduced proportion of heritability explained by the loci identified to date. Given that PRS is a quantitative measure of the individual genetic risk, the more genetic variants are incorporated into the predictive disease risk model, the better the individuals are stratified into the risk subgroups (226). In this scenario, some studies suggest that whole-genome prediction models may account for the unknown genetic risks and, therefore, be able to improve the capacity to predict disease susceptibility, outcomes and treatment response, where the contribution of rare and low-frequency variants will be particularly relevant (223, 239).

On these terms, complex diseases could be comparable to rare disorders, where rare variants with large effect sizes provide disease risk in a small proportion of the population (127, 240). Large-scale sequencing studies will be required to further assess this idea (226, 240).

## Future Perspectives

Despite the large insight provided by GWAS and the admixture mapping scans during the last decades, it remains a large proportion of the missing heritability yet to be ascertained for asthma and related traits (22, 23, 125–127). The future of genetic research in asthma will be driven by NGS approaches, which are expected to significantly increase our knowledge of many other complex diseases (114, 126, 241).

The use of NGS technologies in pulmonary diseases is still emerging (242–246). More specifically, only a few asthma genetics studies have used NGS technologies (242, 243, 246) and large consortia studies are underway (247, 248). Because of its prohibitive costs for large population studies, several strategies have been proposed, such as sequencing the subjects from the extremes of the phenotype distribution (245, 246, 249) or the families where multiple individuals affected (250). The combination of NGS with conventional GWAS approaches has been suggested as another promising strategy (251).

Although the limited knowledge of genetic factors involved in asthma available to date hampers our current capacity to predict disease progression and treatment response (22, 23, 125), the use of genetic information to develop novel therapeutic targets is plausible. For instance, DeBoewe et al. recently found the association of protein-truncating variants with asthma located within widely known asthma susceptibility loci, such as IL33 or GSDMB. This reinforced the evidence suggesting the capacity of the genetic research to find potential asthma therapeutic targets. In fact, as a result of GWAS findings, several drugs targeting IL6R, IL-33, and TSLP are in development or are being evaluated in ongoing clinical trials investigating their efficacy to treat asthma and allergic diseases (22, 252, 253).

## Conclusions

Our knowledge of asthma genetics has been greatly improved over the last decade because of GWAS, revealing a number of novel and firm common risk factors with small effects that overall explain a limited proportion of asthma heritability. Nonetheless, the improvements in high-throughput sequencing technologies and their anticipated cost reductions have the promise to accelerate the transition of this knowledge into the clinical practice and to progressively redirect the field toward an integrative multiomics perspective.

## Author Contributions

All the authors were involved in the conception, hypotheses delineation and structuration of this article, drafted the article, and approved the final version of the manuscript.

### Conflict of Interest Statement

The authors declare that the research was conducted in the absence of any commercial or financial relationships that could be construed as a potential conflict of interest.

## Abbreviations

BDR: bronchodilator response
CNVs: copy number variations
GWAS: genome-wide association study
GWIS: genome-wide interaction study
ICS: inhaled corticosteroids
IL-5: interleukin 5
miRNA: microRNA
NGS: next-generation sequencing
OR: odds ratio
pLoF: predicted loss-of-function
PRS: polygenic risk score
SABA: short-acting β2 agonists
SNP: single nucleotide polymorphism.
